# Roots of wheat and rice maintain gravitropic setpoint angles

**DOI:** 10.1093/jxb/erag286

**Published:** 2026-06-11

**Authors:** Fay Stemp-Walsh, Ryan Kaye, Zoë Kitching, Alex King, Marta Del Bianco, Suruchi Roychoudhry, Stefan Kepinski

**Affiliations:** Centre for Plant Sciences, Faculty of Biological Sciences, University of Leeds, Leeds LS2 9JT, UK; Centre for Plant Sciences, Faculty of Biological Sciences, University of Leeds, Leeds LS2 9JT, UK; Centre for Plant Sciences, Faculty of Biological Sciences, University of Leeds, Leeds LS2 9JT, UK; Centre for Plant Sciences, Faculty of Biological Sciences, University of Leeds, Leeds LS2 9JT, UK; Italian Space Agency, 00133 Rome, Italy; Centre for Plant Sciences, Faculty of Biological Sciences, University of Leeds, Leeds LS2 9JT, UK; Centre for Plant Sciences, Faculty of Biological Sciences, University of Leeds, Leeds LS2 9JT, UK; National Institute of Agricultural Botany, UK

**Keywords:** Auxin, gravitropic setpoint angles, gravity stimulus, rice, root gravitropism, wheat

## Abstract

Root growth angle is a key determinant of root system architecture, nutrient capture efficiency, and therefore yield. Yet the mechanisms governing non-vertical growth in cereal roots remain poorly understood. Here, we investigated if cereal roots maintain gravitropic setpoint angles (GSAs) and the hormonal regulatory processes underpinning GSA maintenance in cereals. Firstly, we found that both wheat seminal roots and rice crown roots actively return towards their original growth angles following displacement, consistent with true GSA maintenance. Next, we show that removal of a stable reference to gravity through clinorotation resulted in a characteristic outward curvature in all root types, indicating the presence of an antigravitropic offset similar to that described in Arabidopsis. Exogenous auxin treatment induced steeper rooting in both species, suggesting conserved hormonal regulatory mechanisms of GSA in both monocots and dicots. Interestingly, lateral root GSAs displayed species-specific differences: wheat laterals returned to their GSAs more effectively than rice laterals, which showed slower and incomplete responses. Together, these findings establish that cereal roots maintain GSAs through gravity-dependent and auxin-regulated mechanisms, providing a novel framework for understanding and manipulating root system architecture in monocot crops.

## Teaser

Cereal roots maintain gravitropic setpoint angles (GSAs) via auxin-dependent mechanisms.

## Introduction

Plant root systems are highly plastic and shaped by the integration of information from their surrounding environment. Plant organ developmental responses guided by directional stimuli are known as tropisms (Muthert *et al.*, 2020). Plants use the constant stimulus of gravity to guide the development of both shoot and root architecture via gravitropism. Root statocyte cells contain dense starch-filled amyloplasts known as statoliths located within the gravity-sensing columella cells of the root cap. In roots of flowering plants, including Arabidopsis, wheat, and rice, the sedimentation of statoliths to the bottom of cells in the direction of gravity triggers the generation of an asymmetric auxin gradient, inhibition of cellular elongation, and, eventually, root bending (Kiss *et al.*, 1989; Ottenschläger *et al.*, 2003).

Although the gravity vector is unidirectional, plant organs can grow at spatiotemporally determined angles, called gravitropic setpoint angles (GSAs), that can be either vertical or oblique with respect to gravity. These angles are actively maintained (Digby and Firn, 1995; Mullen and Hangarter, 2003; Roychoudhry *et al.*, 2013, 2023) meaning that a root displaced from its GSA will respond by returning to its original growth angle through differential growth. Non-vertical root growth is an important adaptation to allow plants to efficiently assimilate resources: roots branch out into the surrounding soil environment to enable the plant to capture heterogeneously distributed soil resources including water, nitrogen, and phosphorus over a large surface area (Lynch, 2013; Roychoudhry *et al.*, 2017).

The monocotyledonous root systems of wheat and rice consist of embryonic roots, nodal roots, and lateral roots that can form from both root types. Wheat root system development starts with the emergence of 3–6 embryonic seminal roots at non-vertical angles (Richards and Passioura, 1981). Unlike the vertical primary root of Arabidopsis, these embryonic roots emerge ‘plagiogravitropically’ or at oblique/non-vertical angles (Rich and Watt, 2013). The root angle of wheat seminal roots is shown to be positively associated with nodal root angle (Manschadi *et al.*, 2008). Nodal roots develop post-embryonically from stem nodes and form most of the mature wheat root system. Lateral roots can initiate from endodermis and pericycle cells in both seminal and nodal roots (Orman-Ligeza *et al.*, 2014). In rice, the embryonic seminal root displays vertical growth, but is short-lived and eventually replaced by the more dominant, crown (nodal) roots (Rich and Watt, 2013). Nodal roots and lateral roots, which can branch off from either the seminal root or the nodal roots, develop at varied angles (Abe and Morita, 1994; Inukai *et al.*, 2005). Whether these non-vertical growth angles in wheat and rice are true GSAs is still to be demonstrated.

A number of root angle mutants and root angle-associated quantitative trait loci (QTLs) have been identified in cereal species (Kirschner *et al.*, 2024). For example, the *DEEPER ROOTING 1* (*DRO1*) and *SOIL SURFACE ROOTING 1* (*qSOR1*) QTLs both act in the regulation of root system architecture in rice (Uga *et al.*, 2013; Kitomi *et al.*, 2020). *DRO1* homeologues have been identified and found to have root tip expression in wheat (Ashraf *et al.*, 2019). *DRO1* and *qSOR1* belong to the *LAZY* family, which play a crucial role in plant gravitropism and the regulation of both shoot and root architecture (Jiao *et al*., 2021). *ENHANCED GRAVITROPISM1* (*EGT1*) and *EGT2* can regulate root growth angle in barley and wheat lines. Plants carrying mutations for either gene show steeper seminal and lateral root angle (Kirschner *et al.*, 2021; Fusi *et al.*, 2022). While this evidence collectively suggests that non-vertical growth angles are genetically set in monocots, true root GSAs must be maintained with respect to the gravity vector.

Over the last decade, studies have demonstrated that, as well as regulating gravitropism, auxin plays a crucial role in maintenance of the GSA. In the model plant *Arabidopsis thaliana*, it has been proposed that two opposite auxin fluxes determine the gravitropic response and an antagonistic antigravitropic offset (AGO) (Roychoudhry *et al.*, 2013). The counteraction of the AGO to the gravitropic response allows for the setting and maintenance of the GSA. In Arabidopsis, the AGO is proposed to be inactive in primary roots, resulting in vertical growth (Roychoudhry *et al.*, 2013, 2023). Primary roots can skew from the vertical due to mechanical interaction between the root tip, rotating about its axis, and the substrate (Vaughn and Masson, 2011). To be considered true GSAs, non-vertical root angles should however be maintained through an AGO or an equivalent mechanism.

Here, we investigate if monocot root systems actively maintain GSAs. We also use simulated microgravity via 2D clinorotation to determine if non-vertical roots possess an AGO and further test the effect of auxin on root GSA in monocots. Our findings provide the first molecular framework for the understanding of mechanisms that regulate non-vertical growth patterns in roots of monocot species.

## Materials and methods

### Plant materials

Wheat ‘Bobwhite’ and rice ‘Nipponbare’ varieties were used for all experiments. Wheat experiments were grown under conditions of 22 °C day, 15 °C night, and a 16 h photoperiod, and rice experiments were grown in 27 °C, with a 12 h photoperiod. Seeds were surface-sterilized for 3 h using chlorine gas seed sterilization. Wheat ‘Bobwhite’ seeds or de-husked ‘Nipponbare’ rice seeds were placed on moist filter paper and cold treated at 4 °C for 2 d before use. Rice seeds were then additionally moved to a 27 °C, 12 h photoperiod for 2 d to allow germination.

### Wheat seminal and rice crown root reorientations

Seeds were placed into Cyg seed germination pouches (Mega International, Minneapolis, MN, USA) with the embryo orientated so that the germ was facing outwards and downwards. Pouches were wrapped in foil to exclude light and placed upright in a reservoir of Hoagland’s No 2 basal salt solution (Hoagland and Arnon, 1938), and plants were allowed to grow for 5 d (wheat) or 2 d (rice). Plants were imaged, reoriented by 30°, and reimaged after 24 h. Seminal root tip angles were analysed before and after reorientation using RootNav (Pound *et al.*, 2013) and measurements were processed using Microsoft Excel and Python. Roots that showed no growth or with a starting angle <30° were excluded from the analysis as reorientation would have placed the roots beyond the vertical (0°) and transposed them to the other side. All images were captured using a Sony Cyber-Shot DSC RX100. Non-reorientated control plants were imaged at the same time as the reorientated plants but were not reorientated.

### Wheat and rice lateral root reorientations

Ten-day-old wheat and rice seedlings were grown on 245 mm (wheat) or 120 mm (rice) square plates in Hoagland’s No 2 (wheat) or Yoshida’s (rice) medium (Yoshida *et al.*, 1971) in their respective growth conditions. Plates were reorientated by 30° and imaged using an Epson Perfection V800 flatbed scanner. Lateral root tip angles measured as the angle from vertical of a 1 mm segment measured from the root tip were analysed with ImageJ for all lateral root experiments. Non-reorientated control plates were imaged at the same time as the reorientated plants but were not reorientated.

### Wheat and rice root clinorotation

A single seedling of each plant was grown either in a 120 mm square plate with Hoaglands or Yoshida medium (for lateral root experiments) or in Cyg seed germination pouches (for seminal root experiments). After 3 d of growth for seminal root systems and 5 d of growth for lateral root systems, plants were placed upon a 1 rpm clinostat in their respective growth conditions. Wheat plants were clinorotated at room temperature and rice plants were clinorotated at 27 °C. The plates or pouches were wrapped in aluminium foil to exclude light from the roots and were clinorotated for 6 h with imaging before and after clinorotation.

### Wheat and rice auxin treatments

Plants were grown as for reorientations but were placed in a reservoir of Hoagland’s No 2 containing indole-3-acetic acid (IAA) (or mock treated) to a given concentration from a 100 mM stock solution of IAA dissolved in 70% ethanol. Wheat plants were photographed after 6 d of growth and rice plants were photographed after 4 d of growth in the pouches. For lateral root auxin treatments, plants were grown as described above, but for 10–12 d to allow lateral roots to develop. Plants were not reorientated and tip angles of wheat seminal roots and rice crown roots were measured with RootNav (Pound *et al.*, 2013).

## Results

### Wheat seminal roots and rice crown roots actively maintain gravitropic setpoint angles

**Fig. 1.**
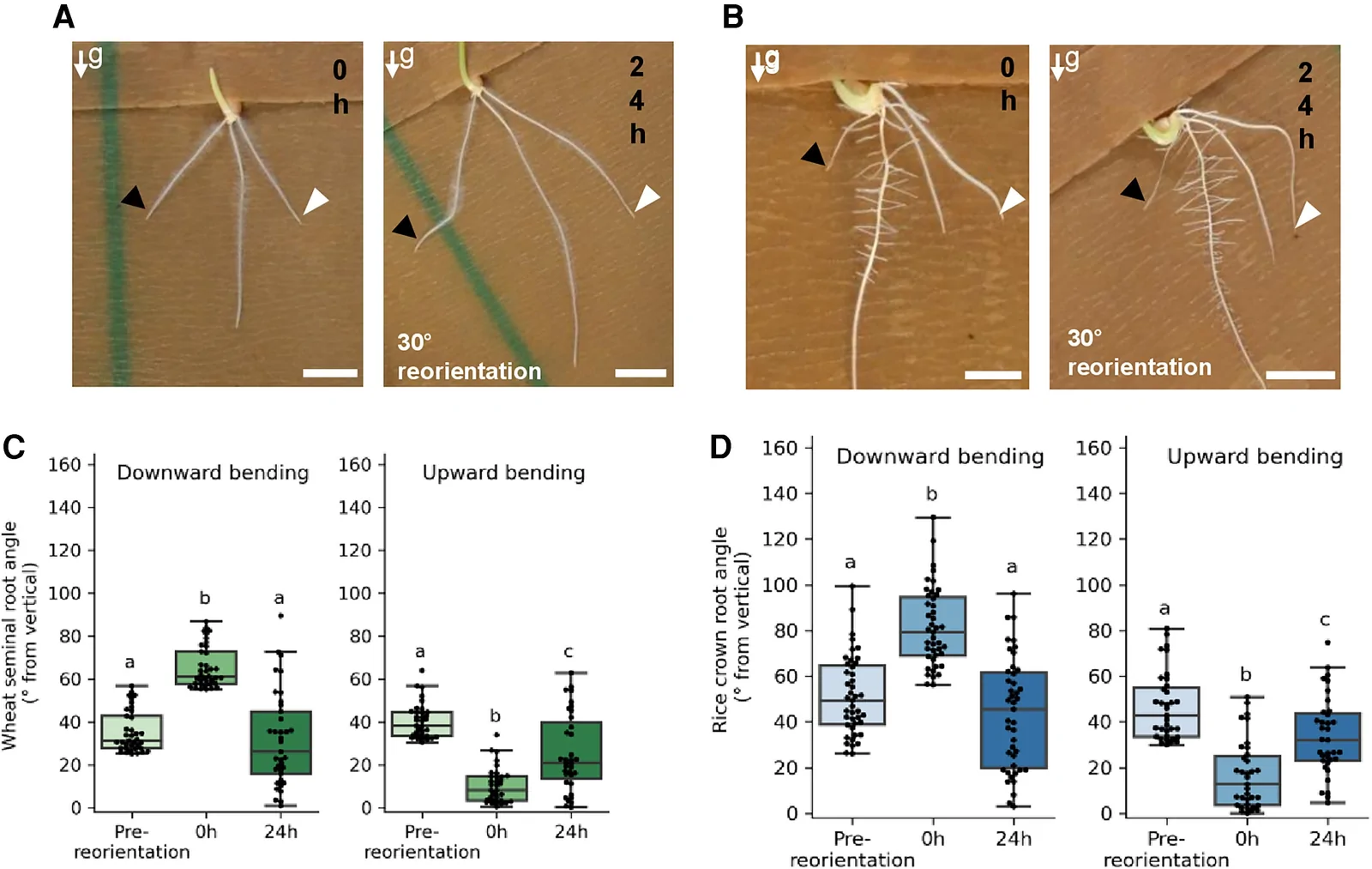
Wheat seminal and rice crown roots return towards their original angle after reorientation. Upward (black arrows) and downward (white arrows) bending wheat ‘Bobwhite’ seminal roots (A, C) and rice ‘Nipponbare’ crown (B, D) roots reorientated at 30° for 24 h. Plants were grown in germination pouches and imaged at 0 and 24 h, ‘g’=direction of gravity, scale bar=10 mm, *P*<0.05.

The first step to establish whether non-vertical growth angles of cereal roots are true GSAs is testing if they are maintained after a change in orientation with respect to gravity. To this aim, we performed reorientation experiments with wheat cv. Bobwhite seminal roots and rice cv. Nipponbare crown roots. Plants were grown vertically and then reorientated by 30° for 24 h, with the root tip angles measured before and after reorientation. A 30° reorientation angle was chosen so roots would not be reorientated past the vertical axis (Fig. 1). A 30° reorientation angle also meant that roots were either reorientated above (downward bending) or below (upward bending) their growth angles. Wheat seminal roots returned to their original angle when downwards bending (Fig. 1A, white arrows, and C). Upwards bending wheat seminal roots bent towards the angle (Fig. 1A, black arrows), but were still more vertical than the pre-reorientation angle after the 24 h period (Fig. 1C). Similarly, rice crown roots also showed faster downwards bending than upward bending (Fig. 1B, D). This could suggest that wheat seminal roots and rice crown roots actively maintain their growth angles, implying that their non-vertical growth angles could fall into the definition of GSAs.

### Wheat seminal and rice crown roots bend outwards under clinorotation

**Fig. 2.**
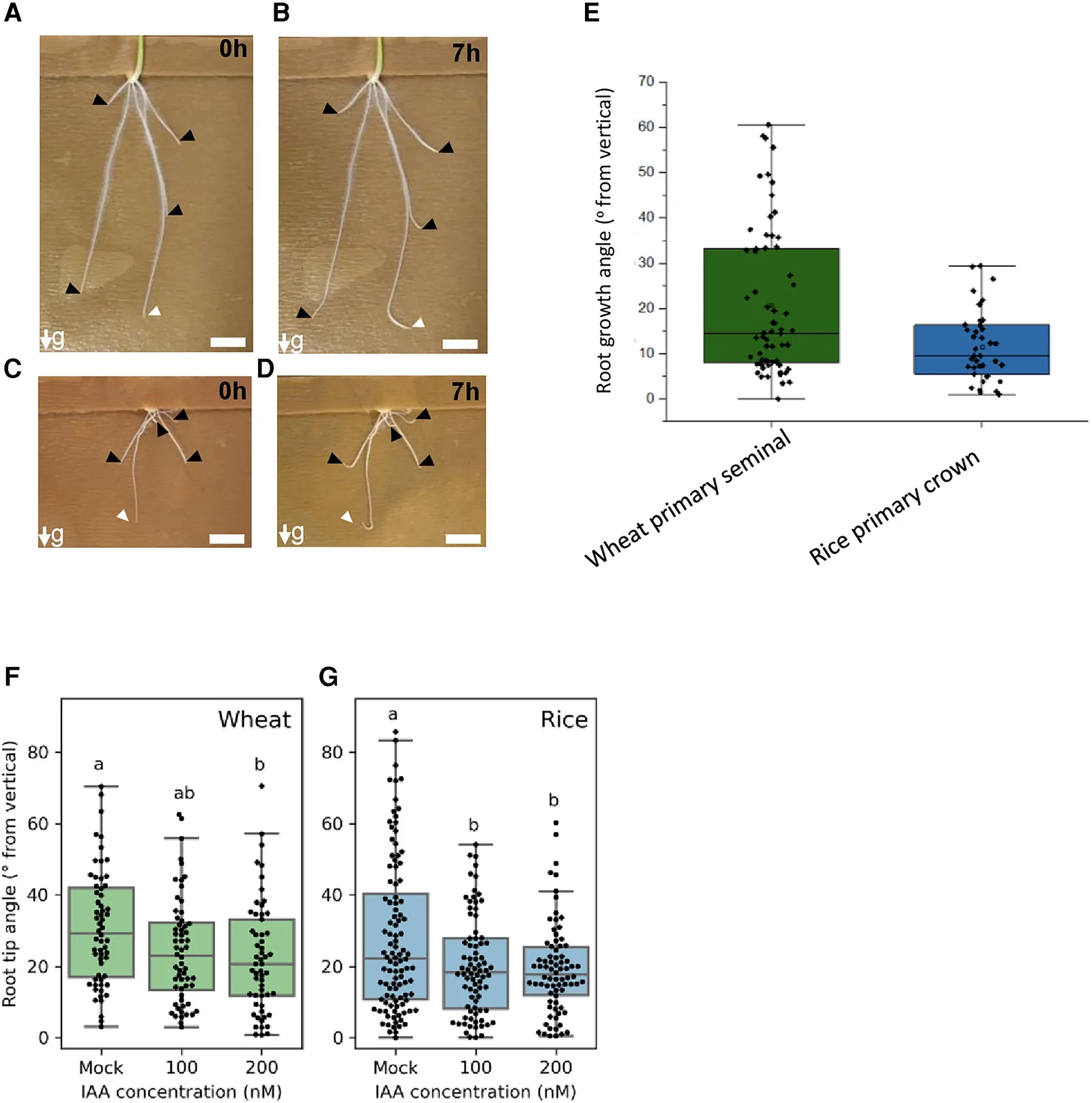
Wheat seminal roots and rice crown roots bend outward upon clinorotation. Wheat seminal roots (A, B) and rice crown roots (C, D) pre-clinorotation (A, C) and root responses to clinorotation at 1 rpm after 6 h (B, D). Arrows indicate root tips, with white arrows showing primary root tips. Scale bars=10 mm, ‘g’ represents the direction of gravity pre-clinorotation. Both wheat and rice lateral roots show significant and characteristic upward and outward bending post-clinorotation. (E) Quantification of primary seminal root and crown root growth angles in wheat and rice seedlings. Wheat (F) and rice (G) root tip angles after treatment with 100 nM and 200 nM IAA, *P*<0.05.

Because the AGO, which balances the gravitropic response, is stable in the time frame of the graviresponse, the removal of a stable reference to gravity, such as by 2D clinorotation, induces a characteristic outward curvature in Arabidopsis lateral roots (Roychoudhry *et al.*, 2013). We therefore used clinorotation (see Supplementary Fig. S1A) to investigate whether wheat seminal and rice crown roots would display the sign of an AGO in response to removal of gravity input. After 7 h of 1 rpm clinorotation, outward and upward curvature of wheat seminal and rice crown roots was seen, similar to what was observed in Arabidopsis lateral roots (Fig. 2A–D, black arrows). Interestingly, in contrast to vertical Arabidopsis primary roots, which do not display any changes after 2D clinorotation (Roychoudhry *et al.*, 2013), vertically growing wheat and rice seminal roots displayed upward bending similar to crown roots (Fig. 2A–D, white arrows). We hypothesize that cereal seminal roots may possess an AGO that enables growth at a slight non-vertical growth angle (Fig. 2E). In this context, it is likely that the three wheat seminal roots arrange themselves as a pyramid, that becomes flattened on the 2D experimental surface (Supplementary Fig. S1B–D).

### Auxin induces deeper rooting in wheat and rice

Non-vertical GSA is regulated, in Arabidopsis seedlings, by the plant hormone auxin (Roychoudhry *et al.*, 2013; Ruiz Rosquete *et al.*, 2013). We therefore tested if auxin was able to influence the root growth angle of wheat seminal and rice nodal roots. We treated wheat and rice seedlings with increasing concentrations of the naturally occurring auxin IAA. IAA treatment made both wheat seminal roots and rice crown roots more vertical (Fig. 2F, G). However, the root growth angle of both species did not change significantly across the range of auxin concentrations tested, indicating that the effect of auxin on cereal root growth angle is not dose dependent in the investigated hormone range. By showing that auxin also regulates root growth angle in wheat and rice, our findings could suggest that similar molecular mechanisms regulate GSA in Arabidopsis and cereals.

### Wheat and rice lateral roots demonstrate differences in gravitropic setpoint angle maintenance

In cereals, both seminal and crown roots can produce non-vertical lateral roots (Kirschner *et al.*, 2024). Initial analysis of lateral root GSAs of wheat and rice showed species-specific differences, with wheat having a steeper GSA than the more horizontally emerging rice lateral roots (Supplementary Fig. S2A, B). To assess whether lateral root growth angles in wheat and rice were also maintained relative to gravity, wheat and rice lateral roots were subjected to the same tests used on wheat seminal roots and rice crown roots.

**Fig. 3.**
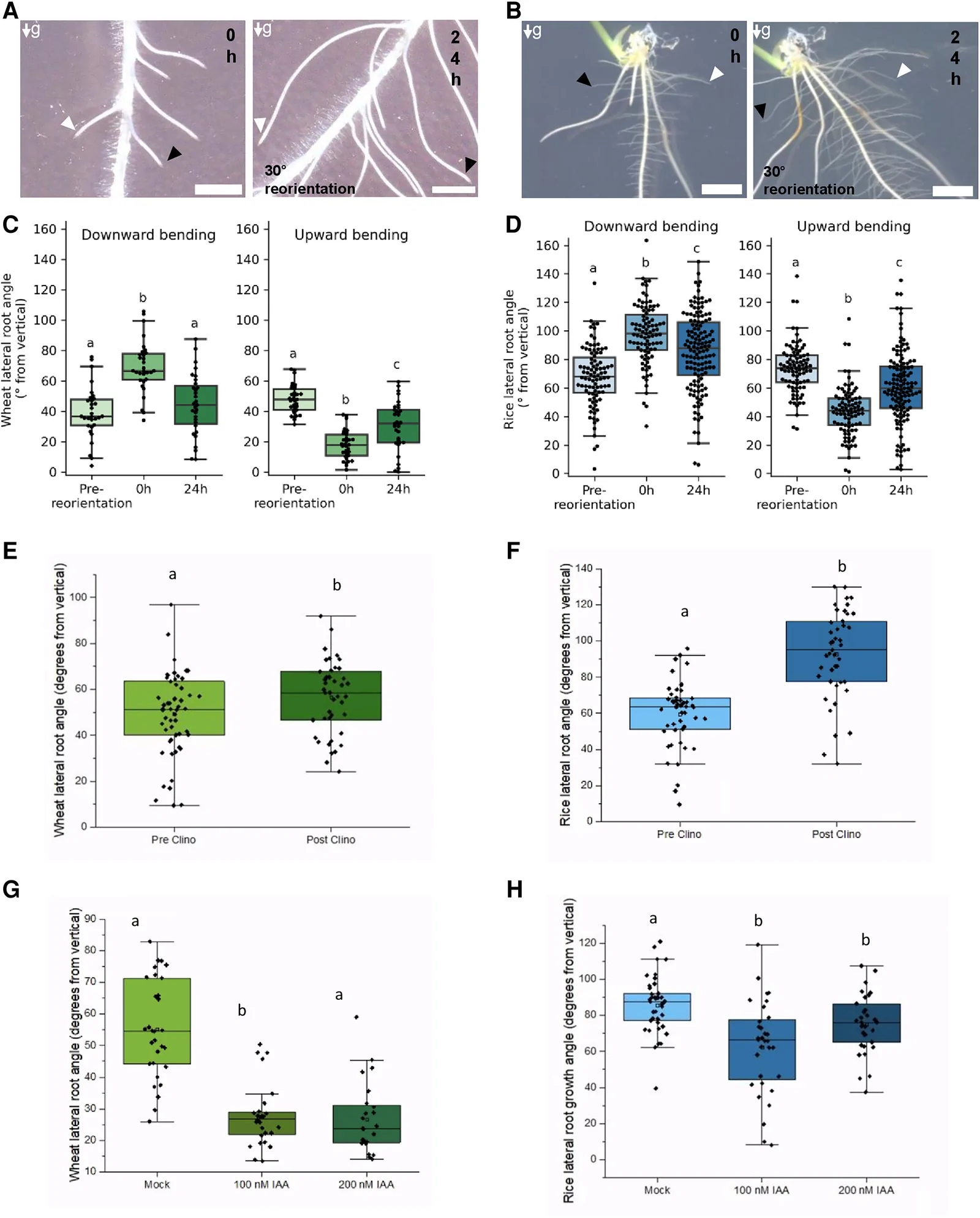
Wheat and rice lateral root growth angle response after 30° reorientation. (A, B) Upward (black arrows) and downward (white arrows) bending in gravistimulated wheat ‘Bobwhite’ (A, C) and rice ‘Nipponbare’ (B, D) lateral roots reorientated at 30° for 24 h. Plants were grown on agar and imaged at 0 h and 24 h, ‘g’=direction of gravity, scale bar=3 mm, *P*<0.05. Change in angle in wheat (E) and rice (F) lateral roots after 6 h of clinorotation at 4 rpm. Both wheat and rice lateral roots show significant and characteristic upward and outward bending post-clinorotation. Effect of auxin treatment on lateral root GSA in wheat (G) and rice (H) lateral roots. Auxin induces steeper rooting in lateral roots of both species.

First, wheat and rice lateral roots were gravistimulated at 30° (Fig. 3A–D). In wheat, similarly to seminal roots, lateral roots showed a faster downward bending and a slower upward bending, and were able to return to their original growth angles or close, respectively (Fig. 3C). In rice lateral roots, instead, both the upward and downward bending responses were very slow, and roots failed to return to their original growth angles after 24 h (Fig. 3D). To assess if longer time would allow cereal lateral roots to return closer to their original GSA, we performed reorientation experiments over 48 h (Supplementary Fig. S3A, B). We found that, in rice, downwards bending lateral roots never returned to their original GSA and only displayed a small change in angle. Upwards bending rice laterals roots, instead, returned closer towards their GSA after 36 h with a wide range in angles (Supplementary Fig. S3B). Interestingly, while both upward and downward bending wheat lateral roots returned to their GSA, after only 12 h from reorientation they became progressively more vertical at successive time points (Supplementary Fig. S3A).

Next, wheat and rice lateral roots were tested for the presence of an AGO and for their responsiveness to auxin in the context of the control of non-vertical growth. Clinorotation was used to remove a stable reference to gravity to investigate whether wheat and rice lateral roots would display the sign of an AGO (see Supplementary Fig. S1A for setup). After 6 h of clinorotation, both wheat and rice lateral roots displayed the characteristic upward bending (Fig. 3E, F). While wheat lateral roots bent upwards to a similar degree to wheat seminal roots (Fig. 3E), the upward bending in rice lateral roots was very prominent compared with rice crown roots (Fig. 3F). Conversely, while the growth angle regulation by auxin in rice laterals was comparable with that of crown roots, growth angles of wheat lateral roots were much more responsive to auxin treatment than any other root type investigated (Fig. 3G, H). Interestingly, while auxin induced steeper rooting in rice lateral roots at both 100 nM and 200 nM, wheat lateral roots became shallower at 200 nM of auxin treatment (Fig. 3G, H). These data highlight the difference in growth angle maintenance not only between cereal species, but also between root types.

### Seminal and lateral roots of wheat and rice gradually adopt more vertical orientations in developmental time

To assess whether any of the observed phenotypes in response to gravistimulation could be dependent on a change in growth angle in time, as observed in Arabidopsis lateral roots (Mullen and Hangarter, 2003), we characterized root growth angle over time in different root types of wheat and rice. Wheat seminal roots and rice crown roots form at similar stages in plant development and emerge at non-vertical angles, in a similar manner to the lateral roots of these species. The root growth angle from the vertical axis was measured for wheat seminal and rice crown roots in 5-day-old plants. The seminal growth angle became gradually more vertical during development, with the angle of rice crown roots being steeper than that of wheat seminal roots (Supplementary Fig. S3C, D). Since lateral roots emerge 6–10 d post-germination in monocots (Wang *et al.*, 2018), plants were grown for 10 d prior to analysis of lateral root development. Lateral roots of wheat became slightly more vertical over time (Supplementary Fig. S3E) compared with rice lateral roots, which maintained a more horizontal angle throughout the time period (Supplementary Fig. S3F).

## Discussion

The establishment of non-vertical growth angles is the main determinant of plant architecture. Understanding the mechanisms of root growth angle control in crop species is therefore important for optimizing root system architecture. Root GSAs have been well characterized in Arabidopsis lateral roots but less so in other monocot species (Roychoudhry *et al.*, 2013, 2023; Ruiz-Rosquete *et al.*, 2013). Here, we found that the non-vertically growing roots of wheat and rice maintain GSAs that change during root development and in response to loss of a constant gravity stimulus or application of exogenous auxin. Our findings provide insight into the mechanisms for cereal root growth angle control and show differences between wheat and rice monocot root systems.

The fibrous root system architecture of cereals differs dramatically from that of the dicot Arabidopsis, so we used the early-forming and non-vertical wheat and rice roots for GSA analysis. Our results showed that all wheat and rice root types investigated became more vertical over a 48 h period, with wheat seminal and rice crown roots becoming steeper in the first 24 h, whereas lateral roots showed an altered GSA between 24 h and 48 h (Fig. 1). Wheat lateral roots had a faster change in GSA over time than those of rice. Wheat lateral roots have an initial steeper GSA than rice lateral roots which were less vertical (Supplementary Fig. S2). Wheat seminal roots have been described to change to a more downwards orientation with distance from the base of the plant which could be when roots reach an older developmental stage. The authors hypothesized that this could be due to internal factors or in combination with external factors controlling the root system development (Nakamoto, 1994). This shows the importance of the genetic and environmental regulation of GSA maintenance and root gravitropic responses. Rice crown roots also show plagiotropic growth, and it has been proposed that the crown root growth angle is determined by its emergence position as well as a potential correlation between root diameter and the gravitropic response (Oyanagi *et al.*, 1993).

Root responses to the change in gravity stimulus from a 30° reorientation differed between the two species. Wheat seminal and rice crown roots both returned towards their GSAs for both upwards and downwards bending roots. Wheat lateral roots returned to their original GSA faster and then became more vertical in comparison with rice laterals which were still returning towards their GSAs after 48 h (Fig. 3A–D). This could be an indicator of a difference in root growth rate or the scale of gravitropic response between wheat and rice root systems. Rice only forms one seminal root, so more horizontal lateral roots may allow coverage of a larger surface area. Comparisons with wheat lateral root GSA maintenance can be made with Arabidopsis lateral roots as both have similar lateral root GSAs which are steeper than those of rice (Roychoudhry *et al.*, 2013), so rice could potentially have more differences in root gravitropic responses. The differences in GSAs and reorientation root growth responses indicate that gravitropic response mechanisms differ within different monocot species as well as in comparison with dicots. Arabidopsis mutants with higher levels of auxin or increased auxin response have more vertical lateral roots than the wild type (Roychoudhry *et al.*, 2013), so it is possible that rice lateral roots could potentially have lower auxin levels or a stronger auxin-dependent AGO than in wheat lateral roots.

Clinorotation results in omnilateral gravitational stimulation, and this loss of a constant gravity reference causes an outwards and upwards bending growth response in Arabidopsis lateral roots (Roychoudhry *et al.*, 2013). Observations following wheat and rice clinorotation found similar root responses in wheat seminal and rice crown roots (Fig. 2). The first wheat seminal root to form (‘the primary seminal root’) usually grows vertically in a 2D growth system as seen here; however, this root was also observed to grow up- and outwards with clinorotation. A study of wheat cultivars grown in the soil in field conditions found that the primary seminal root does not grow vertically but rather at a smaller angle than the other later-forming seminal roots (Nakamoto, 1994), so all wheat seminal roots are likely to have an AGO, unlike the vertically growing Arabidopsis primary root.

A study of plagiotropic or non-vertical wheat seminal roots suggested these to be influenced by the external environment and by internal factors including growth hormones (Nakamoto, 1994). Arabidopsis lateral root GSA is auxin regulated, and auxin treatment causes more vertical lateral root growth (Roychoudhry *et al.*, 2013; Ruiz Rosquete *et al.*, 2013). Arabidopsis lateral roots shift to more vertical GSAs with 50–100 nM IAA treatment, and a similar finding was also seen with bean basal and lateral roots (Roychoudhry *et al.*, 2017). We found that IAA treatment increased the verticality of both wheat seminal and rice crown root GSA (Fig. 3G, H), indicating that auxin is important in GSA maintenance for these cereal species. Auxin may be a factor in the differences seen between the lateral roots of wheat and rice. One initial study of the effects of IAA treatment on wheat seminal roots showed only small effects on root growth angle but a decrease in root elongation growth rate for two different wheat cultivars (Oyanagi *et al.*, 1993). More recent findings in barley cv. Morex showed that barley had significantly steeper seminal root angles following IAA treatment and 90° reorientation (Fusi *et al.*, 2022). Auxin (IAA) application was shown to restore root gravitropic response in rice lateral rootless mutants (*Lrt1*), revealing that auxin is important in rice root gravitropism (Chhun *et al.*, 2003). Studies have shown that wheat and rice have many gene families important for auxin function including *auxin response factor* (*ARF*) genes (Sato *et al.*, 2001; Qiao *et al.*, 2018) and *PIN-FORMED* (*PIN*) genes (Kumar *et al.*, 2021).

Knowledge of root angle control in crop species provides a basis for altering root phenotypes to improve nutrient uptake as different root system ideotypes are proposed to optimize resource capture for water and different nutrients. The ‘steep, cheap, and deep’ ideotype of more vertical root systems can increase nitrogen and water uptake, whereas a shallower topsoil rooting ideotype could improve phosphorus capture (Lynch, 2019). Indeed, maize roots become steeper when grown in nitrogen-deficient field conditions (Trachsel *et al.*, 2013). Moreover, a study of seminal root angles of a Japanese wheat cultivar under controlled conditions showed that northern cultivars were deeper rooting than the southern cultivars which are usually grown under wetter conditions (Oyanagi *et al*., 1993). *DRO1* was first identified in rice and functions in root growth angle control as *DRO1* overexpression leads to more vertical root systems and higher yields in water-limited environments (Uga *et al.*, 2013). In rice, the loss of function of a *DRO1* homologue named *qSOR1* (*quantitative trait locus for SOIL SURFACE ROOTING 1*) resulted in shallower rooting rice which improved yield under saline conditions (Kitomi *et al.*, 2020). Characterization of root system ideotypes and discoveries of root angle-regulating genes in crop species has potential for optimizing root system architecture (Uga, 2021). Understanding of root gravitropic responses in cereal species is important as the ability to alter root systems for environmental adaptation is key for future crop yield increases.

## Supplementary Material

erag286_Supplementary_Data

## Data Availability

All data supporting the findings of this study are available within the article and its Supplementary data. Raw image data underlying Figs 1–3 are available from the corresponding authors upon reasonable request. No restrictions apply to the availability of the data or materials generated in this study.

## Supplementary data

The following supplementary data are available at *JXB* online.

Fig. S1. Clinorotation setup for monocot root systems.

Fig. S2. Wheat and rice lateral root systems.

Fig. S3. Wheat and rice lateral root growth angle responses to 30° reorientation over a 48 h time-course.
