# Supplementary material for: Roots of wheat and rice maintain gravitropic setpoint angles
Source: J Exp Bot. 2026 Jun 11;77(17):5585–93. doi: 10.1093/jxb/erag286 (PMC13577761; doi:10.1093/jxb/erag286)
Supplement: Supplementary file 1 [file erag286_supplementary_data.pdf]

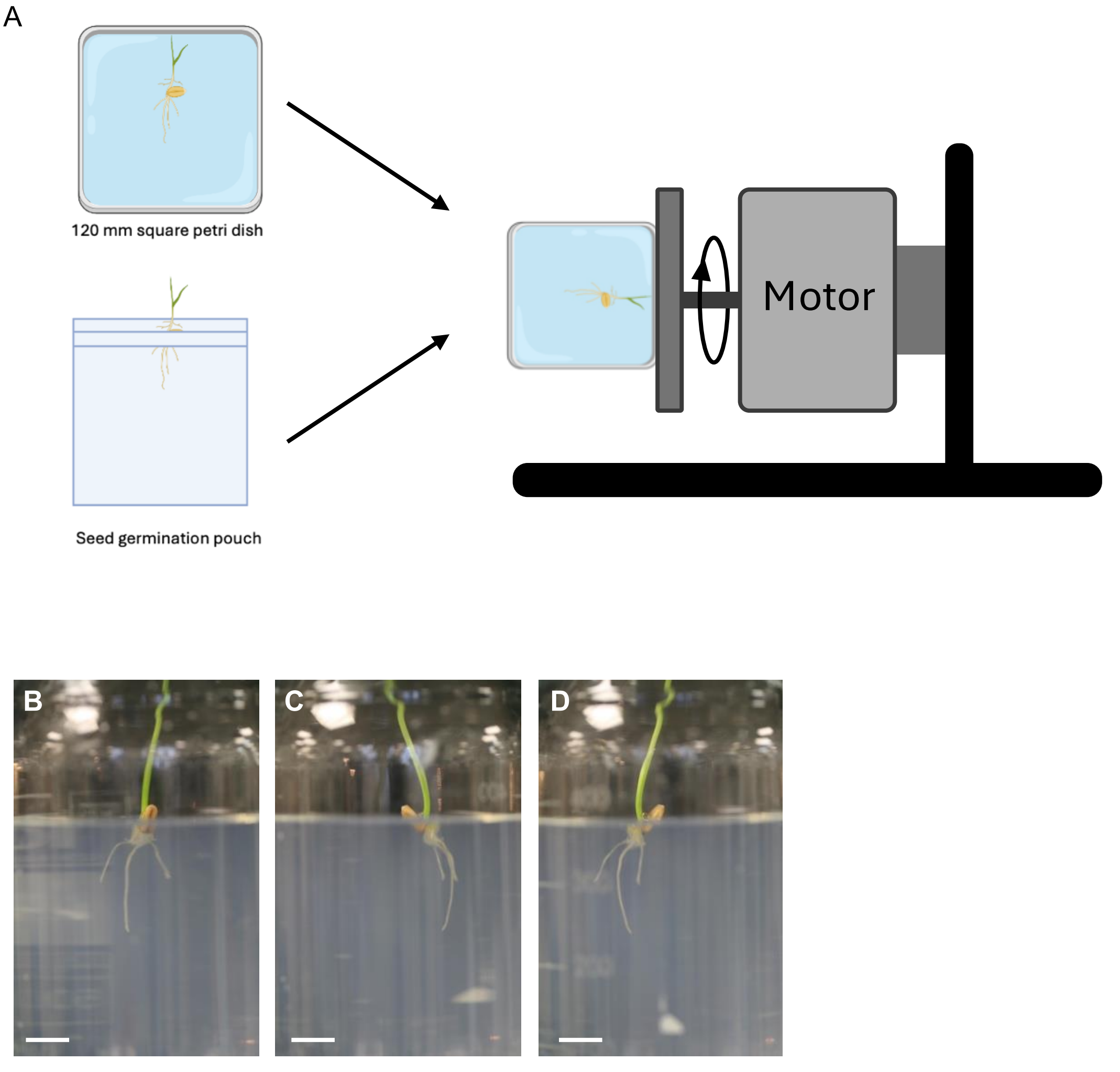

Supplementary Figure 1: Clinorotation set up for monocot root systems.
(A) Schematic representation of clinorotation setup of wheat or rice seedlings. Single seedlings were grown either in 120 mm square petri dishes or on seed germination pouches supplemented with Hoaglands or Yoshidas solid or liquid medium for 3-7 days. A maximum of two plates or seed germination pouches were mounted on the clinostat at a time to ensure that root systems were aligned along the axis of rotation. (B-D) 5-day old wheat seedling grown in Hoaglands No. 2 agar and imaged from different angles. Scale bars = 10 mm.

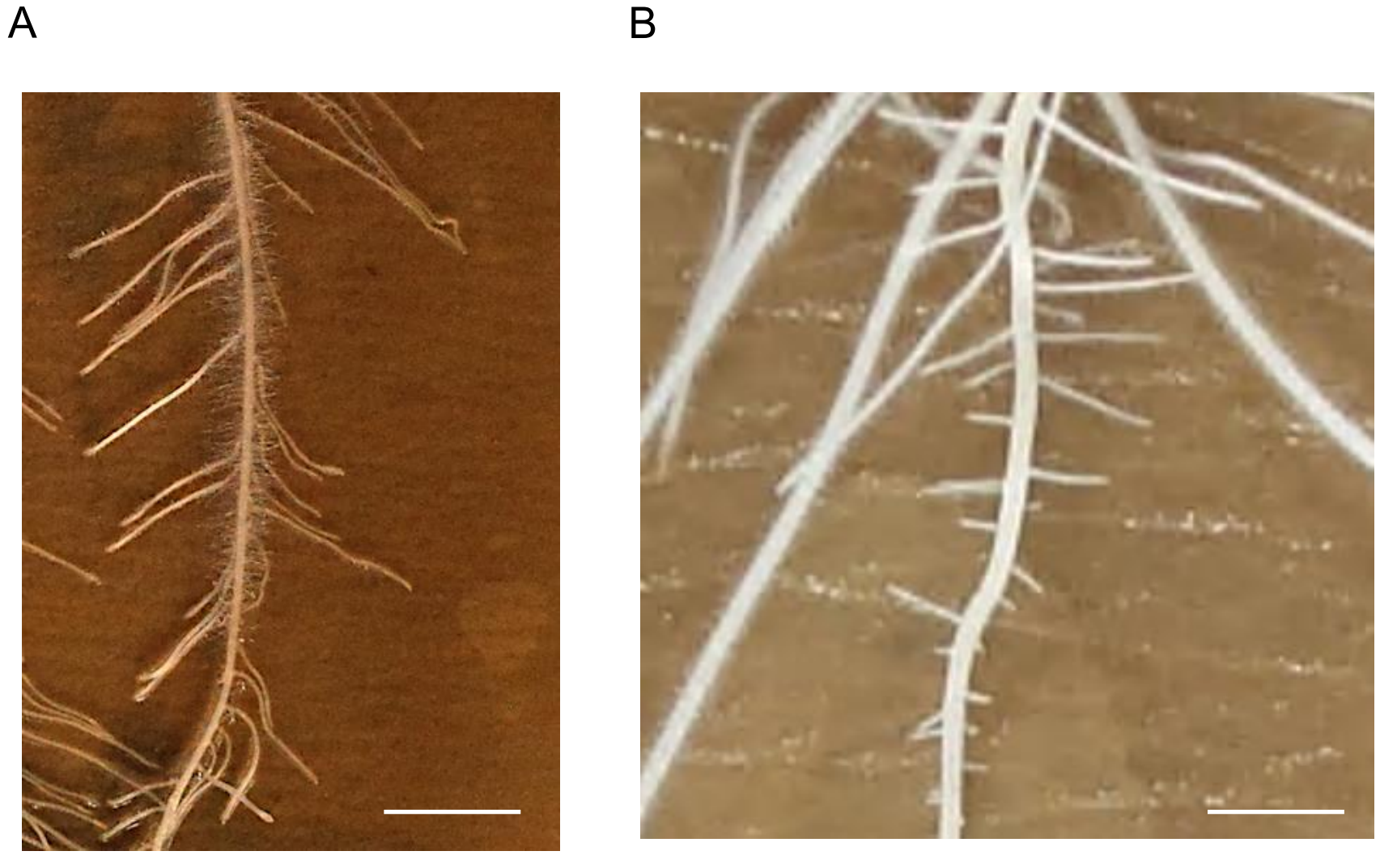

Supplementary Figure 2: Representative images of (A) wheat and (B) rice lateral roots (LRs) from seedlings grown in ‘cyg’ seed germination pouches on Hoaglands medium. Wheat LRs adopt steeper orientations than those of rice. Scale bar = 1 cm.

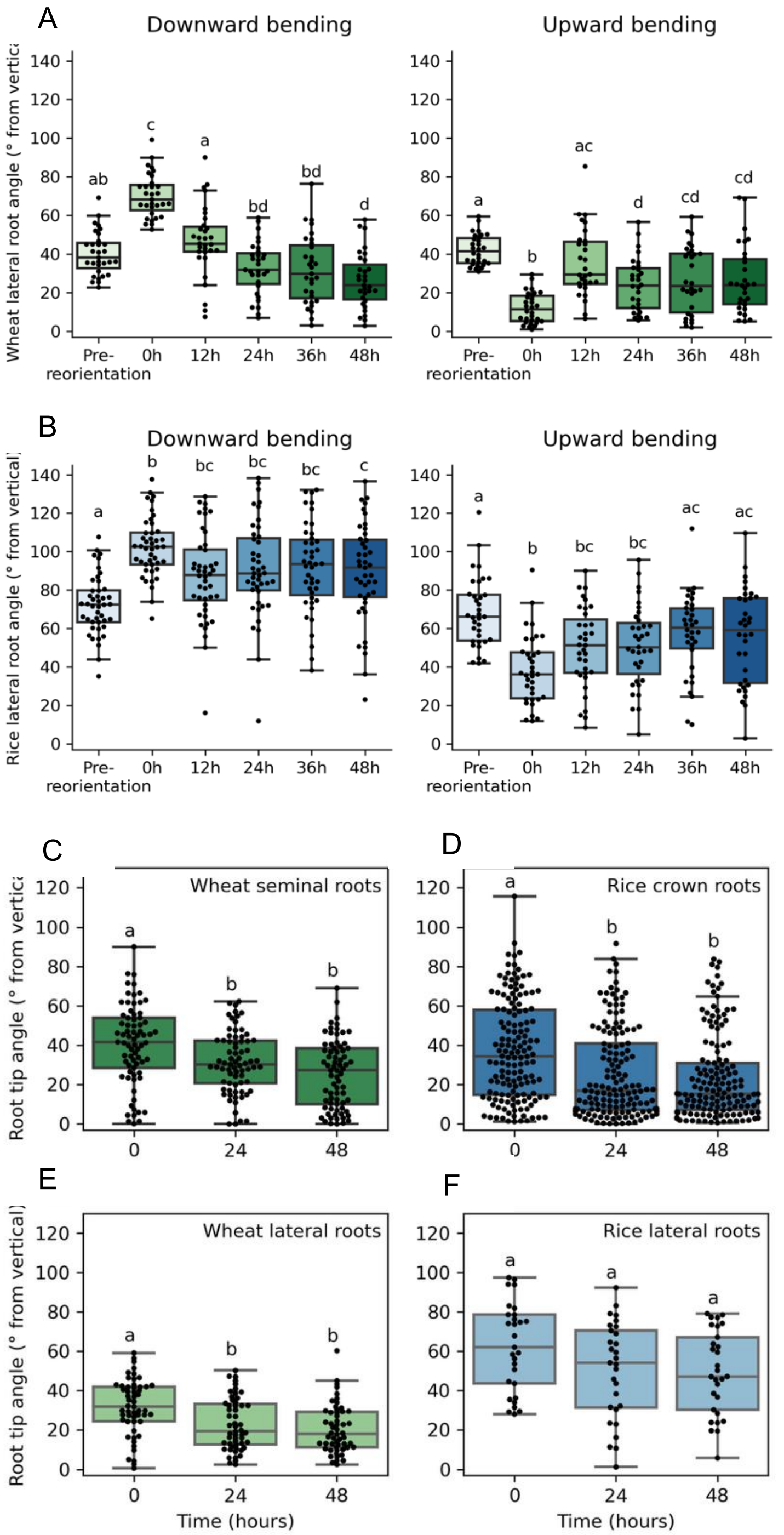

Supplementary Figure 3: Wheat and rice lateral root growth angle responses to 30° reorientation over a 48-hour time-course. Upward and downward bending wheat 'Bobwhite' (A) and rice 'Nipponbare' (B) lateral roots reorientated by 30° for 48 hours. Plants were grown on Hoaglands No. 2 medium with agar and imaged every 12 hours, $p<0.05$. (C-F) Change in wheat and rice root growth angles over time. Wheat seminal (C), rice crown (D), wheat lateral (E) and rice lateral (F) root tip angles over 48 hours, $p<0.05$.
